# “Trying as Hard as I Can”– Narratives of Failure and Success in the Experience of Housing Insecurity

**DOI:** 10.1007/s42087-021-00268-1

**Published:** 2022-02-17

**Authors:** Hannah Wolf

**Affiliations:** Lehrstuhl für Allgemeine Soziologie, Universität Potsdam, Potsdam, Germany

**Keywords:** Housing crisis, Home, Individualization, Governmentality, Liminality, Communitas

## Abstract

The housing crisis represents a liminal experience: a loss of the taken-for-granted and the suspension of ontological security has put individuals in a situation of potentiality in which both conceptions of home and of personal identity are open to transformation. Empirically assessing this liminal transition allows us to understand the refiguration processes of both home and subjectivities. This has both conceptual and political implications: with ongoing individualization of responsibility in virtually all spheres of social life, it is no longer possible to assume that the private sphere of home is an arena in which individuals are free and secured from societal forces, pressures, and compulsions. Instead, we might find ourselves in a transient liminal period in which the very meaning and psycho-social foundation of home are being transformed. To understand these processes is not only an epistemological but also a political endeavor, for only by understanding the psycho-social implications of the housing crisis can we acknowledge its embeddedness in and relation to processes of societal individualization, as well as the potential to open up pathways to the emergence of a liminal *communitas*.

## Introduction

For years, the housing crisis as a widespread and global phenomenon has been on the public agenda. As an object of academic interest, it has been theorized and researched largely from politico-economic perspectives (Aalbers, 2016; Fields & Uffer, 2016; Madden & Marcuse, 2016), human rights perspectives (Fitzpatrick et al., 2014), and critical urban studies (Harvey, 2012; Marcuse, 2009), as well as in terms of gentrification and displacement (Atkinson, 2000; Slater, 2012). A lot of attention, both public and academic, has been given to the emergence of housing struggles from the bottom up: renters’ initiatives and social movements that have formed and collectivized in order to politically and legally fight for a right to the city. All in all, the focus of research lies heavily on (a) structural forces that affect and determine the shape and dynamics of the housing sector and (b) collective struggles and social movement organizations that aim to counter the current power relations and influence these dynamics.

This paper adds to this body of literature by taking a hitherto largely overlooked perspective and pursuing the question of how the current housing crisis and the experiences within it can indeed be viewed as part of an ongoing process of individualization and responsibilization. More specifically, it asks how individuals make sense of and cope with the situation of permanent housing insecurity. By drawing on in-depth interviews conducted over the course of 5 years in Berlin, I explore the subjective narratives, reasons, and emotions that accompany personal experiences of losing one’s home. The analysis shows that, despite the fact that people do — quite rightly — place the responsibility for the housing crisis on structural forces as well as political and economic actors, they simultaneously hold themselves responsible for having failed, or succeeded, under these circumstances. This finding can be interpreted as an indication of a more general process of individualization of responsibility that does not stop at the threshold of the private sphere, such as the home, but indeed extends beyond the public/private divide and becomes internalized, practiced, and thus a part of a process of governmental subjectivation. Furthermore, this process of individualization of responsibility through the experience of housing insecurity is not homogeneous but varies widely.

In the first section, I outline the current housing situation in Berlin and shed light on the processes of privatization, commodification, and financialization as its main structural driving forces. The second section of this paper presents two emblematic and ideal–typical cases of housing insecurity in the city that have been constructed out of the empirical material I collected and presents them as experiences of liminality. It furthermore explores the narratives of failure and success and the accompanying emotions and moral judgments. In the third section, these findings are employed in a conceptual discussion on the experience and ideology of private space. The main argument of this paper is that the personal experience of housing insecurity has the potential to shape both subjectivities and imaginations of home in an enduring way, opening up possibilities for both increasing individualization trends and the emergence of a liminal *communitas*.

## Berlin’s Housing Situation

Berlin still has a reputation of being “a place where one can live well for little money” (Lebuhn, 2015, p. 100). However, a closer look reveals that the city has undergone a thorough refiguration in the past 30 years since its reunification of former East and West Berlin. While Berlin still attracts a large number of people, finding an affordable place to live in the city has become a challenge for both newcomers and established renters. The average rent has more than doubled in 10 years (Statista, 2021), and the number of households who have to spend more than 30% of their income on rent is at about 45%. While for quite some time political and administrative decision makers have ignored this development, it was back on the agenda in 2014 and became part of a political discourse around “housing as the new social question.” There was a somewhat belated process of awareness regarding this problem, and even after it had been politically acknowledged, change was slow. In February 2020, the so-called “Berliner Mietendeckel-Gesetz” came into effect: a law that caps or freezes rents in the private sector at a certain level. This law, which was meant to be effective for 5 years, has been much disputed and debated since its very first discussion in Berlin’s House of Representatives. In April 2021, the law was deemed unconstitutional by the Federal Constitutional Court. Thus, the suspension and temporary relief of financial struggles for a lot of households has ended and Berlin seems to have once more entered the global race to financialize housing.

This development started in the 1990s with the fall of the Berlin Wall, as well as the abolishment of the common public interest legislation on housing (*Wohngemeinnützigkeit*) and the restructuring of the welfare state from welfare to workfare (Aalbers & Holm, 2008) on a national level. The aim and principle of *Wohngemeinnützigkeit* had been to provide large-scale affordable housing in such a way that it compelled housing companies to adopt a non- or low-profit policy in exchange for tax deductions, thus securing moderate rents. By 1990, the overall housing situation had relaxed, so that the political will to uphold this mode of continuous housing subsidies diminished. Abolishing *Wohngemeinnützigkeit* meant that housing companies became regular players on the market, with no special funding or tax relief, so they needed and wanted to generate profits. The second factor, the restructuring from welfare to workfare, affected the structure and distribution of social housing: state subsidies were shifted from buildings to people. The state no longer funded housing companies which then offered renters affordable housing, but instead started to offer individual housing subsidies (*Wohngeld*) to eligible individual citizens. The effect of this shift was a shrinking amount of rent-regulated housing and thus a fundamental change in housing policy: “Instead of supplying broad levels of the population, housing policies are now increasingly limited to targeting marginalised social groups” (Aalbers & Holm, 2008, p. 13).

In Berlin, on a city scale, both these developments came together in a situation of financial strain.

The combination of new regulatory principles on a national level together with the need to consolidate the city’s severely indebted households led to a large-scale privatization or “selling-off of social housing” (Aalbers & Holm, 2008, p. 12). In 1991, there were 19 publicly owned housing companies with a share of 28% of all city-wide housing units; from 1995 onward, more than 200,000 of these public housing units were sold to private housing companies. In less than 15 years, the public housing stock was halved to 15% in 2008.

By 2000, the stock of social housing had been reduced to 72,000, and the selling off continued: this third wave of privatization “could be described as a simple but massive sale of public housing” (Aalbers & Holm, 2008, p. 14). Who were the buyers of these housing stocks? A relatively small number (5%) of apartments were sold to tenants. A much larger part of the housing stock was privatized by turning public housing companies into private housing companies. And by far the largest part of the housing stock was sold to institutional and financial investors, altogether about 58% of all privatized housing. One of the most known and cited examples is the company Cerberus, which bought 65,000 apartments from the public housing corporation GSW in 2004. With the large-scale entry of such investors into Berlin’s housing market, the city entered the global process of financializing housing (Rolnik, 2019). If you had moved to Berlin in the early 2000s, the city you encountered would have been quite different than it was 10 years ago: while there was still a comparatively relaxed rental market, the ownership structure and thus the power relations within this market had changed drastically. A growing amount of housing was owned and provided by large-scale financial investors whose “core business is real estate speculation, leading to a buying and selling of housing units” (Aalbers & Holm, 2008, p. 15). This change in Berlin’s local housing market was tied to larger transnational developments: starting around 1980, the discrepancy between the value of the world’s financial assets and the GDP grew larger and larger, resulting in “super-accumulation” and the “search for internationalization of investments” (Rolnik, 2019, pp. 16–17). There was simply more money made in financial markets, stock exchanges, etc. than could be spent on or invested in products or enterprises in domestic markets. Housing or real estate became the “spatial fix” for this large amount of surplus capital (Harvey, 2001).

The current situation of Berlin’s rental market is difficult to assess, since there is no legal obligation to provide transparency of property ownership. In recent years, the social movement “Deutsche Wohnen & Co enteignen” has emerged, fighting for the socialization of large-scale housing companies. The initiative “Wem gehört Berlin?” (“Who owns Berlin?,” Der Tagesspiegel, 2019) has gathered information about the ownership of real estate with the help of Berlin’s renters, who were asked to provide information about the owners of their respective places of residence. By way of this “swarm intelligence,” the initiative has contributed to a greater degree of transparency and knowledge about the city’s property landscape. They find that, in contrast to other German metropols, 60% of Berlin’s flats are owned by actors and companies in the private economy, that is, economic actors whose business model consists of generating profits from flats, either by renting or trading them. Fifteen percent, which equates to 230,000 housing units, are owned by large companies listed on the stock exchange, such as the aforementioned Deutsche Wohnen, Vonovia, ADO Properties, Covivio, Akelius, TAG Immobilien, and Grand City Properties. The value of their shares has exploded since 2012, with Vonovia quadrupling its value, and Grand City Properties even noting an increase of 700% share value. This increase was made possible due to four main factors: the demographic development, i.e., migration into the city; scarcity of housing leading to rent increases; the lack of modernization, which means that investments in modernization gain large returns; and lastly the overall financial situation offering loans with low interest rates. As “Wem gehört Berlin?” states in a report:

“This results in a spiral because the value of a property is measured by the quality of the building together with the expected development of rents in the building. So the interplay between low interest rates, modernization and opportunities for rent increases is worth its weight in gold. Houses cost more every year. And so the big companies can show high increases in the value of their houses on their balance sheets every year. Those who understood this in time were able to accumulate fantastic sums.” (Der Tagesspiegel, 2019)

What are the effects of this development for renters in Berlin? Berlin’s population has been increasing steadily since 2005, with a total of 343,489 individuals moving into the city between 2011 and 2019 (IBB – Investitionsbank Berlin, 2020). More than 2 million households are currently residing in Berlin, 53% of which have only one member and 28% of which are households consisting of two people. Half of the households have a monthly income of more than 2000 Euros, while one-fifth has to make ends meet with less than 1300 euros. These numbers are not distributed evenly across Berlin; there are, as in every city, districts with a higher average income, such as Steglitz-Zehlendorf and Pankow, and those with lower average income, such as Neukölln and Lichtenberg. But, even for vulnerable groups, being eligible for social housing does not automatically translate into living in social housing developments. With the amount of social housing stock steadily declining since 2010 with 150,000 to currently 95,723 units, there is an obvious shortage of affordable und publicly funded housing. On the other hand, both sale prices and rents are increasing, with newly built buildings and flats in inner city districts demanding the highest prices. The decrease in social and affordable housing means that a growing number of people are competing to find a place to live, and those who have found affordable housing are less likely to move. With nearly half of Berlin’s tenants fearing that they will soon not be able to pay their rent, housing insecurity has become a common experience (Die Zeit, 2020).

## Housing Insecurity and Liminal Experiences of Home

Statistics can provide an overview and general trends, but in order to understand the heterogeneous experiences of Berlin’s housing insecurity, a more in-depth approach is necessary. It is especially important in this context to distinguish between housing and home. Home here is understood as an assemblage consisting of material and symbolic aspects and entities, a range of interrelated and interdependent actants, and importantly practices, emotions, and affects that take place within and indeed constitute this web of material-symbolic-actor networks (Blunt & Dowling, 2006; Brickell, 2012). Instead of assuming that home is a fixed place that is simply there, the production of home needs to be understood as a fluid and emergent process. At the same time, home does indeed prove to be fundamentally important to human beings. Home — whatever it may be and wherever it may individually be situated — provides people with a sense of ontological security, through everyday routines as well as through the experience of continuity between the past, present, and future (Bachelard, 1987; Douglas, 1991; Giddens, 1991). Home is thus intricately linked to identity. What we consider to be our home has to do with who we consider ourselves to be, how we construct our biographies, and how we make sense of ourselves in the world (Casey, 2009; Easthope, 2004). Home is thus a deeply personal matter. However, as such it is also socially shaped. As social beings, we are never independent or autonomous of influences, contexts, and the web of societal factors (in the broadest sense) in which our lives are embedded. Individuals themselves are entangled within the assemblage of home; their subjectivities both shape and influence and reciprocally *are* shaped through practices, meanings, materialities, and imaginations of home. It has been widely argued that the growing retreat from the public sphere and the late-modern “obsession with home” (Atkinson & Jacobs, 2016, p. 44) reflect a “wide-spread form of social privatism in which the home, because of social, economic, and technological changes, has taken on an increasing primacy in the lives of people” (Atkinson & Jacobs, 2016, p. 37). In line with this, home has become both the material and imaginal site of a “retreat from collectivity” (ibid.). Following Rose (1999), we can connect this process to larger governmental strategies of “disciplining the soul” by fostering a rejection of “modes of collective belonging in favor of an ill-defined individualism” (Atkinson & Jacobs, 2016, p. 38).

Our social embeddedness and entanglement at home is largely unconscious in our everyday lives, as long as we can habitually reproduce daily routines without irritation or disruption, or as Tuan (1980, p. 4) calls it, being “at home in an unselfconscious way.” However, moments of irritation can occur that provoke and foster the emergence and reflection of a conscious “sense of place” (Easthope, 2004, p. 133):

“This is important because when the places in which our habitus is enacted are changed rapidly by external forces (such as those increasing in our era of globalisation and escalating uncertainty) the possibility of a feeling of rootedness diminishes, and our need to create a sense of place as ‘secure and stable’ is heightened” (Easthope, 2004, p. 133).

Housing insecurity, whether it comes as a sudden shock or as a creeping and persistent feeling of fear, represents one of these rapid changes that foster a conscious sense of place: “the financialization of housing strongly influences the everyday experience of home and, therefore, leads to different emotional and affective dimensions and notions of home” (Pohl et al., 2020, p. 2). It forces individuals to reflect on the nature of and their relationship to what exactly it is that they call home; it — at least temporarily — puts an end to an unselfconscious feeling of rootedness and simultaneously opens up a space of contingency and potentiality. Such a situation can be conceptualized as an experience of liminality. Liminality denotes the feeling of being “betwixt and between” (Turner, 1977, p. 95) and was originally coined by anthropologist van Gennep ([1909] 1960) in regard to the tripartite pattern of rites of passage — rituals in which members of a community change their social status, i.e., from adolescence to adulthood, through marriage and childbirth, and through death and burial. Van Gennep detected a supposedly universal structure, “a ritual form” (Thomassen, 2009, p. 6) of such rites, and more generally of processes of transition and transformation, consisting of separation, transition, and incorporation. Within this sequential pattern, he called the middle stage — the transition — the liminal period, which is arguably the most complex and important part of the ritual sequence. Liminal, stemming from the Latin word *limen*, refers to a threshold that cannot be crossed easily or unconditionally. At the *limen*, we are forced to linger, to wait. Liminality refers to spatial and temporal in-betweenness: we are temporarily cast outside of the social structure, yet expect to be re-incorporated into it. One enters a social space in which a former status is lost, waiting to acquire a new one. A transformation is required. Liminality is as such a truly spatio-temporal and experiential concept, which can only be observed as it develops: “Liminality makes sense only within social dramas as they unfold” (Thomassen, 2009, p. 13).

Liminality as a concept is not limited to ritual contexts but can be found in western, modern experiences as well:

“individuals and whole communities can find themselves thrust into the chaos of circumstances in which the usual order of things is disturbed, ruptured, shocked or destroyed, and these events can vary from collectively experienced floods, earthquakes and riots to more local phenomena like divorces, job-losses and significant deaths. Such are the slings and arrows of outrageous fortune. These are experiences in the true sense of the word: they are things we must go through. They are also things that mark and transform us: we are different when we come out the ‘other side’” (Stenner, 2018, p. 24).

Applying this idea to the phenomenon of housing insecurity and home as an assemblage of material, symbolic, and identity-related aspects as sketched above, it becomes apparent that the current housing question or housing crisis is both an individual and collective experience of disruption and transformation. As an individual experience, it is indeed a situation that might alter one’s own understanding and imagination of home; it is a social drama that both unfolds and inhibits the possibility of radical change and transformation. As a collective experience, it is by no means homogeneous but needs to be considered as a varied process of altering shared understandings of home.

Considering that one’s sense of place and personal identity are intrinsically interwoven, and that liminal experiences require transformation, I argue that to think with liminality is an opportunity to explore and understand processes of subjectivation, individualization of responsibility, and governmentalization. Conceptually, there is a gap between what is commonly described as structure and agency: we assume that changes on a structural macro-level impact agencies, identities, and practices on a micro-level, but through what modes exactly this takes place is still disputed in the social sciences (Ritzer, 2000). As Thomassen (2009) has argued, liminality might offer the “missing link” between these related yet incommensurable analytical entities. My conceptual argument, therefore, is that it is the disruption and suspension rather than the reproduction of social order and routine that functions as a motor of *both* social change and social continuity. As Stenner (2018, p. 178) stresses,

“liminality points precisely to situations of potentiality in which […] ‘what happens’ might take many different courses, but the actual outcome is uncertain. Liminality is about the process of becoming and not about explaining what already exists.”

Looking beneath the structural level of housing policies, distribution, and accessibility, the remainder of this paper is concerned with the different psychosocial transformations that this experience entails. It investigates people’s reflections on what exactly it is that they call home, and thereby offers an entry point for reflection on and analysis of the interconnections between home, identity, habitus, and practice. In the context of this paper and this special issue on individualization of responsibility, I shall focus on the different modes in which people make sense of this situation of insecurity, attribute responsibility for it, and narrate their biographies and identities in regard to it.

## Notes on Methodology and Research Design

From 2016 to 2021, I conducted multiple in-depth narrative interviews with 20 individual households who either currently were or had been in a situation of housing insecurity. Participants were recruited through various channels, including social media, social work/housing assistance institutions, personal contacts, and housing activist groups. The sampling strategy was aimed at generating heterogeneity in terms of socio-economic background, gender, ethnicity, and age, as well as in terms of the individual experience of housing insecurity. Thus, the lived realities of the participants in my study run the gamut from actual homelessness, i.e., rough sleeping, to institutionalized temporary accommodation, private arrangements of co-housing and subletting, and tenancies with regular rental agreements. The logic underlying this heterogeneous sampling is the acknowledgement that housing insecurity has become a common experience among a large share of Berlin’s residents, despite individually different circumstances. While both the feeling of housing insecurity and the feeling of home are deeply personal and subjective experiences, the study’s aim was to uncover any identifiable common structures and dynamics of this experience. Semi-structured narrative interviews were conducted, whenever possible at the participant’s current place of residence, as well as — due to the outbreak of the COVID-19 pandemic — via videoconferencing and walking interviews (Evans & Jones, 2011). Following a Grounded Theory approach (Glaser & Strauss, 1967; Timonen et al., 2018), I aimed for a constant and iterative interplay between data collection and data analysis and interpretation, thus allowing the initial findings to guide further research, while later findings could contradict and modify the initial assumptions. Employing the qualities of liminality as sensitizing concepts, central categories and concepts emerged that connected spatio-temporal experiences with transformative processes of identity and subjectivation, for example, in realms of agency and responsibility, as well as emotions such as shame and pride. In the following section, I present two cases in sufficient detail to explore processes of responsibilization and governmental subjectivation. These cases represent both paradigmatic and maximum-variation individual cases (Flyvbjerg, 2006) of the experience of liminality through housing insecurity in Berlin. They are selected “on the basis of their information content” (Flyvbjerg, 2006, p. 230) rather than on the basis of representational and generalizable qualities. In both cases, the unconscious sense of place experienced and routinized through everyday practices is disrupted, resulting in a conscious transformation of aspirations, imaginations, and practices. The cases are paradigmatic in the sense that they both signify how the experience of liminality is a process in which governmental subjectivation unfolds. At the same time, they vary in important dimensions, such as age, class, duration of tenure, and rootedness, as well as the intensity of the disruption and uprooting, in order to better flesh out “the significance of various circumstances” (Flyvbjerg, 2006, p. 230).

## Subjectivation Processes at Home

### You Wait and Look and Have to Stay Flexible

Paul and Sabine,^1^ a couple in their early 30 s, had been living together in Berlin’s Friedrichshain district for 5 years. One morning, they woke up to an unfamiliar whirring sound. Looking out of the window, they saw a drone flying up and down the façade of the house in which their flat was situated on the fourth floor. Immediately, Paul tells me, he thought that this was not some teenager’s prank but a sign that the building was up for sale. He turned out to be right when they followed this hunch, searched online for their address a few days later, and found multiple advertisements for individual flats — one of them quite clearly the one he and his girlfriend were currently calling their home. Soon after that, they received a letter in the post informing them that their flat was up for sale. They were offered the opportunity to purchase the flat themselves, for an amount of money they could not afford. One sentence stood out: “Nothing will change for you.” The letter suggested that most potential buyers would have little to no intention of actually living in the flat themselves, so what Paul and Sabine, as well as all their neighbors, were facing was simply a change of ownership. Soon after they received the letter, they were contacted by a real estate agent. Somebody was interested in the flat; a viewing appointment had to be arranged. Paul tells me, they thought this would be the beginning of a long row of viewings and awkward situations with strangers in their private rooms. However, things changed quickly when the very first prospective buyer, a man in his 20 s from Austria, decided he wanted to purchase the flat. Furthermore, he did indeed want to move to Berlin. Paul and Sabine had 9 months to move out. Things were about to change for them.

When asked how they felt about and coped with the situation, Paul talks a lot about anger and aggression. He frequently uses the analogy that as a prehistoric man he would have just beaten and killed anyone trying to intrude into his home. Being the modern and civilized man that he is, he instead articulates strong feelings of injustice: “The flats are for people who live in the state and pay taxes, and that the state doesn’t protect our flat and says this will definitely not be a speculation object, that’s not right.” He feels a strong sense of abandonment by public institutions and political actors on whom he places the responsibility to secure them the right to housing. Home, in his view, has changed from a place that offers protection to a place in need of protection. However, Paul appears unwilling to actively protest against this perceived injustice. When I ask him whether he had joined any of the multiple demonstrations and initiatives that have formed around the housing question in Berlin, he tells me “that would be wasted energy because I don’t have any opportunities to do anything to defend myself.” The case for his flat is lost, he explains. Thinking about the future, he articulates ambiguous feelings: on the one hand, there is an intensifying notion of general insecurity and mistrust — “There is this thought, if we move now, who can guarantee that the same won’t happen with the next flat?” — on the other hand, he tells me that having to move out now also provides them a chance “to do things better next time.” He is well aware that the housing situation is worse for other people without a well-paid job such as his, so there is also a moral judgment involved in the sense that as long as he is able to take care of himself autonomously it is his obligation to do so.

With his new job he recently started, he now has the opportunity to put money aside in order to buy a house in the future. Although he explains that living in a small house in the suburbs is not the lifestyle he really wants, he sees no alternative. At the same time, he stresses that “we don’t even know where my partner is going to work next year… so, you wait and look and have to stay flexible.” In the context of individualizing responsibility, these words take on a complex meaning. The contradictory ambiguity between being stripped of control over his own dwelling situation and the assertation that he indeed has control over the situation and can affect it through “better,” more strategic, and, at the same time, more flexible behavior points toward a process of governmental subjectivation, understood here primarily as technologies of the self or “ways in which human beings come to understand and act upon themselves within certain regimes of authority and knowledge, and by means of certain techniques directed to self-improvement” (Rose et al., 2006, p. 11). Governmentality or the “conduct of conduct” (Foucault, 1982) consists in setting conditions and “arranging things so that people, following only their own self-interest, *will do as they ought*” (Scott, 1998, p. 202). This by no means implies an explicit government program aimed at coercing people to act or feel in a certain way, but rather needs to be understood as an assemblage “pulled together from an existing repertoire, a matter of habit, accretion, and bricolage” (Li, 2007, p. 276). In this view, Berlin’s housing situation and the liminal experience of housing insecurity is itself an assemblage in which subjectivities are formed and modified through technologies of the self, such as “autonomy, self-actualization, self-realization, and self-esteem” (Madsen, 2014, p. 814; see also Lemke et al., 2000). Importantly for Paul, the solution that will allow him to obtain control over his own living situation is a modification of his behavior, aspirations, and desires: over the course of our encounters and interviews, he undergoes a transformation towards a responsibilized, self-sufficient yet flexible home-owner.

### I Have to Make Sure This Does Not Happen to Me Again

Christine is a woman in her late 50 s, divorced, long-term unemployed, and with a history of mental illness. She was evicted from her flat in Charlottenburg, in which she had lived for over 20 years, due to rent arrears and subsequently lived in different types of temporary accommodation all over the city for a period of 3 years: first in two different women’s emergency shelters, then in a flat shared with other clients of the social housing emergency system. As a person dependent on state benefits for many years, she is used to financial precarity, to being in contact with street-level bureaucrats, and to the humiliating interactions this can entail. In the context of housing and home, however, these humiliating and exhausting encounters have increased and intensified. She shares a flat with two other women; each of them is appointed an individual social worker who comes by to inspect the flat and meet with them once a week. She tells me there is hardly any privacy. She does not like one of the women living there; there are a lot of conflicts, so Christine has turned her room into her fortress. The door is always locked; she does not leave the room and only quickly sneaks out to go to the toilet. For her, living in this shared temporary accommodation does not resemble living together, but rather living against one another. Tensions arise, which aggravates the stress level and impacts her mental health. Furthermore, social services expect her to find a flat and move out. She is only eligible for a 6-month stay in the flat. In a city with an immensely heated housing market, she desperately needs to change her dwelling situation.

Asked how she feels about and copes with the situation, Christine starts talking about bureaucratic procedures and the interpersonal contact they entail. Like many others, Christine is a client in a network of multiple public and private institutions within the realm of the social assistance system (Gerull & Merckens, 2012). Throughout our communication, she regularly tells me about the difficulties associated with this: she needs to see different caseworkers for different issues, and the exchange of information between them is problematic, resulting in her feeling hassled and pressured instead of supported, and as though she were an object constantly there to be operated on and modified. Christine is aware that what is expected of her is success in finding a new flat, and she reiterates that she is “trying as hard as I can.” With the staggering rent prices, her meager benefit payments, and the virtually non-existent social housing opportunities, she has hardly any chance to find a place to live by herself. She has looked into multi-generational living (*Mehrgenerationenhäuser*) and housing cooperatives (*Wohngenossenschaften*) because, despite the difficulties in the shared temporary accommodation, she likes the idea of living together in a community. However, all these forms of communal living require some kind of entry fee which she cannot afford to pay. When after nearly 3 years in temporary accommodation she has managed to find a small flat for herself, Christine looks exhausted. She tells me about several conversations with the social workers assigned to her case:

“I said, ‘Dear Ms. [X], dear Ms. [Y], dear Ms. [Z], I am not here for you, you are here for me. This means: I do what I do as best as I can. You have to do it better’. […] And then when I came back from the housing assistance office with all my paperwork for the new flat, I told Ms. [X] that the caseworker there had said that I should be proud of myself because I did all this by myself. And Mrs. [X] said ‘But I did it too’.”

Christine gets emotional and angry while narrating this incident. She goes on to explain that she has the impression that the social worker, Ms. [X], expected gratitude and appreciation from her. But Christine insists that she has succeeded all by herself, and indeed she has contacted housing companies, handed in application forms, and finally found an affordable flat without assistance from her social workers. And despite her exhaustion, she really appears to be proud of herself. She tells me, “everyone is telling me that two years ago they would never have thought that I would come out of this phase safe and sound, or even alive.” With regard to liminality, her phrase “come out of this phase” is interesting because it points towards an understanding that the in-between period has actually come to an end. Now that she is no longer officially categorized as “in need of housing” (*wohnungslos*) and has once again entered into a formal rental contract, one can argue that this represents the post-liminal phase of re-incorporation, as van Gennep described it. What about the transformation that liminality requires? Christine explains that she is no longer the person she used to be, that it is hard for her to remember and empathize with her former self. While she refused to take on responsibility for the eviction that happened 3 years ago, she now feels responsible for her present and future. Arriving at this point has been difficult and painful for her, but at the same time she stresses: “I mean, this has cost the state so much money, so now it has to be worth it, you know?” While she clearly articulates that the social help system has failed her, she feels a strong obligation not to be a disappointment to the authorities or to herself. Similar to Paul’s and Sabine’s case, there is a simultaneity of subjectivation and autonomization. Christine is taking care of herself, has started seeing a therapist, and has also become a member of an urban gardening group. “I have to make sure this does not happen to me again,” she tells me.

## Concluding Discussion

These cases are two individual and highly condensed narrated stories of housing insecurity in Berlin. The narration focuses on the ways in which subjectivities and identities were shaped and modified in and through liminal phases. The first section of this paper underlines that these psycho-social processes are embedded in wider political and economic contexts. Through the lenses of liminality and governmentality, the home becomes a powerful site/assemblage in and through which responsibility becomes individualized. In both cases, the spatial imaginary of home turns into a site of personal improvement and achievement. What are the implications of these findings? I want to outline two conceptual points and one political argument:

*First*, the notion of home as a place of refuge and privacy needs to be modified into an understanding of home as a space and a set of practices through and in which governmental subjectivities are shaped and thus the tension of subjectivation and emancipation can be experienced and observed. Blunt and Dowling (2006), among others, have argued strongly against a conflation of house and home and for an understanding of home as a “relation between material and imaginative realms and processes” (Blunt & Dowling, 2006, p. 254). These processes “constitute identity, whereby people’s senses of themselves are related to and produced through lived and metaphorical experiences of home” (Blunt & Dowling, 2006, p. 256). In this view, neither home nor identity are fixed entities but are rather bound together in a reciprocal process of creation, (re)production, and transformation. Housing insecurity posits a threat to spatial imaginaries of home that stabilize identities; it represents an irritation and a loss of the taken-for-granted, to which individuals are prompted to react. In the cases presented here, the ways in which the participants make sense of both the situation and of themselves within this situation can be described as subjectivation in a Foucauldian sense, meaning that “subjectivation confers a form of autonomy on the subject” (Ong-Van-Cung, 2011, p. 151). The subject is compelled to take on responsibility, to act, and to exert agency. This process of responsibilization is simultaneously one of subjugation and autonomization: it “could result in an ‘empowerment’ or ‘responsibilization’ of subjects, forcing them to ‘free’ decision-making in fields of actions” (Lemke, 2002, p. 54). Contrary to the common assumption that home is a place of retreat from such processes of governmentalization, a space that provides security and freedom from societal compulsions, we need to add home to the spheres in which subjects produce themselves as governmental and responsibilized subjects.

*Second*, the idea of the public/private divide, deeply embedded in the commodification of housing, fosters the ongoing individualization of responsibility. The dominant, politically, and culturally reproduced imaginary is one that connects home with privacy (Mallett, 2004). This paves the way for an emerging understanding of home as a site not only of personal fulfillment and self-actualization, but also of personal achievement, as exemplified in the empirical narratives. Likewise, housing insecurity and losing one’s home can become recoded as personal failures. Two possible implications can be drawn from this finding: first, that we can observe an intrusion of the ‘outer’ world into the private sphere, that the “ways of organizing the world, including technology and commodification, are eroding people’s ability to make home, to create a place that is sacred, separate from society and full of significance” (Blunt & Dowling, 2006, p. 14). Second and alternatively, we can conclude that home has never been this private place detached from society but is constituted through an inherent openness and interrelatedness with wider political, social, economic, and cultural processes. Still, the notion of home as the epitome of the private sphere is reproduced in both cultural as well as individual imaginaries of home. Again referring to Foucault, this can be understood as an element of a *dispositif* in which the notion of privacy serves as a powerful governing technology, constituting and reinforcing relations of power (Blunt & Dowling, 2006, p. 256).

*Third*, thinking with liminality offers a vantage point as well as both an empirical and conceptual tool for the close observation of such subjectivation processes as they unfold. It also provides a perspective on political and practical implications. Liminality underlines the openness and potential infiniteness of social processes of emergence and transformation, and thus emphasizes the possibility of change, or indeed the contingency inherent in all social processes. In the context of my research, it has served as a tool to investigate individual psycho-social transformations that at first glance seem to contradict the broader and more public emergence of collective housing struggles, social movements, and “a right to the city” initiatives. While these movements emphasize collectivity, the loss and re-making of home remains a strong individual experience. Housing insecurity is becoming a more and more common and widespread phenomenon, putting ever more people into individually experienced and emotionally charged situations of “betwixt-and-between.” As a liminal experience, it is thus both collective and subjective. In such situations, Turner speaks of the emergence of a liminal *communitas*: a mode of connectivity and interrelatedness that “breaks in through the interstices of structure, in liminality; at the edges of structure, in marginality; and from beneath structure, in inferiority” (Turner, 1977, p. 128). The liminal *communitas* is formed of individuals who share a common existential experience and thus encounter each other as fundamentally equal. Status hierarchies and other properties of social differentiation are temporarily suspended, and it is here that the possibility of radical change, of questioning the social structure *per se*, emerges (Förster, 2003). However, despite the growing extent of housing insecurity, the social imaginary of home as a private space/place appears to be limiting this potentiality of a *shared* liminal experience, and thus the potential for radical change. As Easthope states, when “the possibility of a feeling of rootedness diminishes […] our need to create a sense of place as ‘secure and stable’ is heightened” (Easthope, 2004, p.133). There is thus a dialectic tension in housing struggles that is rarely articulated: the tension between a collective demand for a right and access to housing, and a subjective desire for a place called home (Pohl et al., 2020). Such desire proves to be a powerful force, potentially compelling individuals to transform or adapt their conduct and their aspirations. While in the arena of political demands and claim-making collectivity is voiced, on an individual level the personal desire for one’s own home remains or even intensifies. The political argument of this paper is thus that the varied notions of home need to be included more in debates, claim-making, and political decisions associated with the housing crisis. Actors advocating for a change in the housing system need to address the emotive and affective dimensions of home, beyond the reproduction of home as a private space of refuge and comfort. They need to open up spaces in which those who experience individual liminality can interrelate and connect with others. They need to create opportunities in which feelings of loss, shame, and personal failure can be articulated and shared. As Pohl et al. (2020) stress, “As long as we equate homes and housing, we lose sight of this personal dimension.” The realization that it is not only an issue of financialization and commodification, but is also about a shared experience of desiring a home, can be a powerful way to build a liminal *communitas*: “This relationship is always a ‘happening’, something that arises in instant mutuality, when each person fully experiences the being of the other” (Turner, 1977, p. 136). Only then can the liminal experience unfold its potential to truly transform existing social configurations (Förster, 2003).

Work on governmentality and individualization of responsibility tends to have a rather pessimistic outlook: the shaping of neoliberal subjectivities is often depicted as disintegration and fragmentation of a social bond (Rose, 1996). In the course of this paper, I have tried to show how these processes are interwoven in the space, affects, and imaginatories of home. Home can be viewed as an important site and mode for technologies of the self. However, the notion of a desire for a home as a shared human condition makes it possible to think not only of individual responsibilization but also of collective empowerment. The liminal experience can be a “site of deprivation” and a “space of resistance” at the same time (hooks, 1990). As this paper has argued, the idea of home as the epitome of privacy is but a social construction. Home, as a material and symbolic assemblage, is not detached from society, and the private/public divide is not naturally given, but socially produced. As such, it can be unmade, renegotiated, and the boundaries between individuality and collectivity can be redrawn in different ways. The collective liminal experience can provide the precondition for such a transformation.

## Data Availability

Interview transcript available on request.

## Footnotes

1 All names are pseudonyms.
